# Fine motor control of the jaw following alteration of orofacial afferent inputs

**DOI:** 10.1007/s00784-016-1939-4

**Published:** 2016-08-27

**Authors:** Abhishek Kumar, Eduardo Castrillon, Mats Trulsson, Krister G Svensson, Peter Svensson

**Affiliations:** 1 Section of Oral Rehabilitation, Department of Dental Medicine, Karolinska Institutet, Alfred Nobels allé 8, Box 4064, 141 04 Huddinge, Sweden; 2 SCON | Scandinavian Center for Orofacial Neurosciences, Huddinge, Sweden; 3 Section of Orofacial Pain and Jaw Function, Department of Dentistry, Aarhus University, Aarhus, Denmark

**Keywords:** Local anesthesia, Periodontal mechanoreceptors, Bite force, Variability, Optimization

## Abstract

**Objective:**

The study was designed to investigate if alteration of different orofacial afferent inputs would have different effects on oral fine motor control and to test the hypothesis that reduced afferent inputs will increase the variability of bite force values and jaw muscle activity, and repeated training with splitting of food morsel in conditions with reduced afferent inputs would decrease the variability and lead to optimization of bite force values and jaw muscle activity.

**Material methods:**

Forty-five healthy volunteers participated in a single experimental session and were equally divided into incisal, mucosal, and block anesthesia groups. The participants performed six series (with ten trials) of a standardized hold and split task after the intervention with local anesthesia was made in the respective groups. The hold and split forces along with the corresponding jaw muscle activity were recorded and compared to a reference group.

**Results:**

The hold force and the electromyographic (EMG) activity of the masseter muscles during the hold phase were significantly higher in the incisal and block anesthesia group, as compared to the reference group (*P* < 0.001). However, there was no significant effect of groups on the split force (*P* = 0.975) but a significant decrease in the EMG activity of right masseter in mucosal anesthesia group as compared to the reference group (*P* = 0.006). The results also revealed that there was no significant effect of local anesthesia on the variability of the hold and split force (*P* < 0.677). However, there was a significant decrease in the variability of EMG activity of the jaw closing muscles in the block anesthesia group as compared to the reference group (*P* < 0.041), during the hold phase and a significant increase in the variability of EMG activity of right masseter in the mucosal anesthesia group (*P* = 0.021) along with a significant increase in the EMG activity of anterior temporalis muscle in the incisal anesthesia group, compared to the reference group (*P* = 0.018), during the split phase.

**Conclusions:**

The results of the present study indicated that altering different orofacial afferent inputs may have different effects on some aspects of oral fine motor control. Further, inhibition of afferent inputs from the orofacial or periodontal mechanoreceptors did not increase the variability of bite force values and jaw muscle activity; indicating that the relative precision of the oral fine motor task was not compromised inspite of the anesthesia. The results also suggest the propensity of optimization of bite force values and jaw muscle activity due to repeated splitting of the food morsels, inspite of alteration of sensory inputs.

**Clinical relevance:**

Skill acquisition following a change in oral sensory environment is crucial for understanding how humans learn and re-learn oral motor behaviors and the kind of adaptation that takes place after successful oral rehabilitation procedures.

## Introduction

Over the past couple of decades, there has been extensive focus on understanding the peripheral and neural mechanisms of mastication [1–3]. The trigeminally innervated orofacial tissues contain primary afferent nerve fibers which terminate as receptors (e.g., mechanoreceptors), in different sensory organs. The brain, by virtue of these orofacial mechanoreceptors, assimilates and organizes all the sensory information for sensorimotor regulation and optimize oral functions [4]. Previous studies have suggested that one important class of receptors for sensorimotor regulation and oral fine motor control are the periodontal mechanoreceptors (PMRs) [5–7]. The PMRs provide important information regarding temporal, spatial, and intensive aspects of force acting on the tooth, during the initial tooth food contact [7–12]. Further, this ability for spatially controlling the jaw is disrupted during periodontal anesthesia [5]. Hence, it was suggested that early afferent information about the tooth food contact would contribute to the regulation of the spatial (three-dimensional) control of the jaws.

When first performing novel motor tasks, the muscle activation used to achieve the objective of the action does not typically use the muscles available in the most efficient manner [13]. Motor training resulting from the repetition of a novel motor task results in increased performance with increased representation of the trained muscle in the motor cortex [14, 15]. The primary face motor cortex is said to be important not only for sensorimotor integration and learning of new oral motor skills but also for adaption to alteration in orofacial environment [1, 3]. In the past, studies have demonstrated dramatic changes in the organization of somatosensory cortex following removal of afferent inputs or manipulation of sensory inputs in primates [16]. Therefore, it is suggested that the face sensorimotor cortex can also undergo neuroplastic changes in relation to acquisition of motor skills, or adaptive processes following alterations in intraoral sensory inputs [1, 3]. Several microneurographic studies of the face and oral mucosa have suggested that innervation of individual nerve territories vary considerably. These studies suggest that the perioral region and the tip of the tongue are areas with a particularly high density of mechanoreceptive innervation [17, 18]. Therefore, altering different orofacial afferent inputs may have different effects on oral motor control yet, only a few studies have demonstrated the effect of manipulation of sensory afferent inputs on oral motor performance during oral fine motor tasks [5, 19].

It appears that the use of selective blocks of sensory inputs and its effect on masticatory motor behaviors has been extensively studied in animal models [20]. However, the use of anesthesia to block (reduce) specific intra- and perioral sensory inputs and its effects on oral fine motor control in humans has not been thoroughly explored. Hence, the present study was designed to investigate if altering different orofacial afferent inputs would have different effects on the perturbation of oral fine motor control and jaw muscle activity. Further, we also wanted to test the hypothesis that reduced/no afferent inputs from the orofacial/PMRs will increase the variability of bite force values and jaw muscle activity and repeated training with splitting of food morsel will decrease the variability and lead to the optimization of bite force values and jaw muscle activity, in spite of the reduced inputs from the orofacial/PMRs.

## Methods

### Study participants

Forty-five healthy volunteers (22 men) in the age range of 19–40 years and mean age of 24.0 ± 0.6 (SEM) years, participated in the study. The study participants were university students recruited through Aarhus University’s research participation system and were compensated a minimum of DKK 100/h for their participation. At the time of the experiment, the participants were in good general health with normal healthy dentition, with no known history of periodontal disease. The participants also exhibited a normal occlusion without any gross malocclusion and without increased overjet/overbite or open bite/deep bite of the anterior teeth. The participants were also free from any previous/ongoing endodontic, prosthetic, or orthodontic treatment of the anterior teeth. A temporomandibular disorder (TMD) screener questionnaire ruled out the presence of any TMD or associated symptoms prior to the participation [21]. The study was conducted in accordance with the Declaration of Helsinki II and approved by the ethics committee, Midjutland region, Denmark. Informed consent was obtained from all the participants prior to the start of the experiment.

### Study protocol

The volunteers participated in a single experimental session of approximately one and a half hour. The volunteers were randomly assigned to one of the three groups (*N* = 15), on the basis of intervention made by the examiner (see local anesthesia). These three groups were named as the *mucosal anesthesia group* (mean age 24.2 ± 1.3; six men), the *incisal anesthesia group* (mean age 24.0 ± 0.8; eight men), and the *block anesthesia group* (mean age 23.8 ± 1.0; eight men). The randomization was done using computer-generated codes. During the experimental session, the intervention (in the form of local anesthesia) was made at the start of the experiment and subsequently, the participants were asked to perform six series comprising of ten trials of an intraoral fine motor behavioral task. The behavioral task was demonstrated by the examiner and the participants were not allowed any practice trials before the start of the experiment. The participants were given a break of about 3–5 min after each series. Thus, in a single experimental session, the participants in total performed 60 repetitions of the behavioral task, after the intervention was made.

### Behavioral task

The behavioral task was to hold and split a test food object (flat-faced, bevel-edged, tablet diameter 8 mm, thickness 3 mm, weight 180 mg, Hospitalsapoteket, Aarhus, Denmark) with the anterior teeth [22]. The participants were seated comfortably on an office chair with their hand resting on a table, placed in front of them. The participants held a bite force transducer with one hand while the other hand was used to carry the test food object to the bite force transducer. The participants placed the transducer end along with the test food object horizontally on their lower central incisor (either right or left) and were asked to hold the test food object between their antagonistic upper central incisors. The participants were also asked not to use more force than necessary to control the test food object. Further, after about 3–4 s, the participants were asked to split the test food object [5, 7, 23]. Throughout the experiment, the participants were instructed to use the same teeth, i.e., either right or left central incisor along with antagonistic opposing teeth. The force required to hold and split the test food object during the behavioral task along with the corresponding electromyographic (EMG) activity of the jaw muscles were measured using custom-made analyzing software (Split force analyzer, Klarsen, Denmark).

## Armamentarium

### Local anesthesia

The oral mucosa was anesthetized (mucosal anesthesia group) by intraoral application of a topical local anesthetic gel (lidocaine hydrochloride 2 g). A generous amount (approx. 6–8 g) of the gel was dispensed on the tongue and the participants were asked to spread the gel all over their mouth including the buccal/labial vestibule, gums, and palate, with their tongue. The participants were also asked to hold the cream in their mouth for about 5 min and later, rinse their mouth with water. In the remaining two interventions (i.e., incisal anesthesia group and block anesthesia group), injections of local anesthesia (mepivacain 10 mg/ml without vasoconstrictor) were made with a 27-gauge needle. Incisal anesthesia was achieved by local infiltration of about 1-ml local anesthetic solution in the buccal sulcus opposite to the corresponding upper and lower central incisors (unilaterally, either right/left upper and antagonistic right/left lower central incisor). The block anesthesia group was given a bilateral inferior alveolar nerve block, a bilateral lingual nerve block, and a unilateral local infiltration (using the technique mentioned earlier) to the upper central incisors. Thus, about 1.6 ml of the local anesthetic solution was deposited using the conventional standardized inferior alveolar and lingual nerve block injection technique after identifying the anatomical landmarks (coronoid notch, pterygomandibular raphe, and occlusal plane of the mandibular teeth). Clinical anesthesia of the teeth and other areas were assessed by the lack of response to light pressure/touch to the teeth, gingiva, and lip. The participants also reported the effect of anesthesia in the subject-based reports before the first series of the behavioral tasks were performed. The participants were asked to only use the anesthetized teeth when performing the behavioral task.

### Bite force

The “hold forces” and “split forces” during the behavioral tasks were measured using a custom-made strain gauge-based bite force transducer (Physiology Section, IMB, Umeå University, Umeå, Sweden). The participants with their preferred hand, held the 6-cm-long aluminum tube connected to two duralumin blocks, which terminated as two rectangular metal plates [22, 24]. The test food object was placed on the free end of the transducer, which was made with a plastic covering (2 mm) to prevent any potential damage to the teeth. The lower plate was also made with a plastic covering with an indentation to allow proper positioning of the transducer onto the lower teeth. The transducer system has been previously used and was designed in such a way that force measurements would be insensitive to the point of force applied onto the plate [22, 24]. The transducer had an analog output of 12 mV/N and a delay in force to analog output of 2.2 ms.

### EMG activity

The EMG activity corresponding to the force measurements were measured using disposable, bipolar, surface electrodes (30 × 21 mm recording area, 720-01-K, Neuroline, Ambu®, Denmark). The masseter and anterior temporalis muscles were palpated by asking the participants to clench their teeth. A pair of bipolar electrodes were placed 10 mm apart from each other on the central part of the muscle, midway between the anterior and posterior borders and the superior and inferior borders of the right and left masseter muscle (MAL and MAR). A similar pair of electrodes were placed on the anterior part of the left anterior temporalis (TAL) muscle, lateral to the eyebrow. The suprahyoid (SHD) muscle were palpated by asking the participant to swallow and a set of electrodes were placed close to the anterior belly of digastric (SHD) muscle, one (bipolar) electrode on each side (i.e., right and left side on the anterior belly of digastric) 1 cm medial to the base of the mandible at the level of the first and second molar [22, 24, 25]. The skin over the recording positions was thoroughly cleaned with sterile wipes (isopropyl alcohol 70 %). The electrodes were positioned on the skin perpendicular to the direction of the muscle fibers. A common reference electrode was attached to the left wrist of the participant. The EMG was recorded in 10-s epoch. The EMG signals were processed and quantified as root-mean-squares (RMS) values and amplified 10,000 times (Disa 15C01, DK), filtered in the bandwidth 20 Hz to 1 kHz, sampled at 2 kHz and stored for offline analysis [22, 24].

### Data analysis

**Fig. 1:**
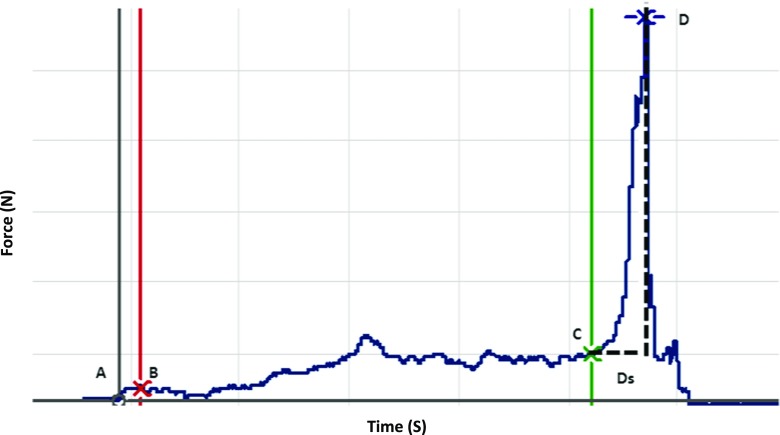
Example of a force profile obtained from a single “hold and split” task. *X-axis* represents the force (N) and *Y-axis* represents time (s). The specific points of interest are *A* initial contact with the test food, *B* 0.2 s after the initial contact with the test food, *C* onset of the split defined as the point at which the force rate exceeded 5 N/s, *D* split force defined the peak force prior to the moment the test food split, indicated by a rapid decrease in the force. *Ds* duration of split defined as the time taken from the onset of the split (*C*) to the actual split (*D*). The hold force was defined as the mean of force exerted from points *B* to *C* and split force was defined as the absolute force at *D*

The data was analyzed using an office computer with customized software. Force and EMG measurements were obtained and checked individually for specific points of interest during each trial. A typical force profile obtained during a single hold and split task is shown in Fig. 1. The hold force was defined as the mean force during the time interval 0.2 s after the initial contact with the test food object (*B*) to the onset of split force (*C*) [11, 22]. The onset of the split phase was defined as the point at which the force rate exceeded 5 N/s, the minimum rate of increase that could be reliably detected in a single trials [11, 22]. The split force was defined as the peak force (*D*) prior to the moment the test food split, which was indicated by a rapid decrease in the force. The duration of the split phase (Ds) was defined as the time required from onset of the split force (*C*) to the peak force (*D*) [11, 22, 24]. The EMG activity of the masticatory muscles were recorded and adjusted for time lag to the corresponding force values [26].

### Statistics

It was decided to compare the data from the present study with a *reference group*. The data for the reference group was obtained from the previous published study in which the exact same procedure of a hold and split task was performed by 20 healthy participants (mean age = 25.7 ± 0.8; ten men), 60 times; without the influence of anesthesia. The data was analyzed for normal distribution with Shapiro–Wilk test and histogram plots. The distribution of the variables was skewed to the right and, therefore, these variables were log-transformed. The log-transformed data thus obtained were analyzed with a two-way analysis of variance (ANOVA) model with repeated measures to analyze the different outcome parameters. The factors in ANOVA were groups (four levels; i.e., mucosal anesthesia, incisal anesthesia, block anesthesia, and reference groups) and series (six levels; i.e., 1–6). Post hoc tests were performed with Tukey honestly significant difference test for multiple comparisons. The variability expressed as coefficient of variation (CV) was calculated as the ratio of standard deviation and mean from 10 trials from each series. A *P* value of <0.05 was considered statistically significant.

To examine the robustness of the scores (variables) obtained from the present study in comparison to the reference group the log-transformed data were converted into *Z* scores. *Z* scores are statistical measurements of a score’s relationship to the mean in a group of scores. The mean in this case was obtained from the previous published study (reference group) in which the exact same procedure of hold and split task was performed by 20 healthy participants [18]. The *Z* score were calculated using the formula; *Z* score = individual variable (present study) − mean (previous study)/standard deviation (previous study). The *Z* scores in the range of −1.64 and +1.64 means that the *P* value associated is with a 90 % confidence interval (CI) and is significant at a level of 0.10. A *Z* score in this range (i.e., −1.64 to 1.64) in context of the present study would mean that the perturbations are subtle, whereas a value <1.64 or >1.64 would indicate a more robust change/perturbation.

## Results

The participants in all the three (anesthetized) groups were able to complete the six series of the hold and split task without any technical problems. The participants confirmed the presence of subjective symptoms related to local anesthesia after the interventions were made. The subject-based reports on the frequencies of the effect of anesthesia at different intraoral sites are tabulated in Table 1. The force profiles obtained during the task were similar in the three groups. Nevertheless, there were some interesting differences between the groups and across the six series, which are presented in the following section.

**Table 1:** Self-reports on the frequency of occurrence of the effect of anesthesia in the three groups on the various orofacial sites after administration

	Mucosal anesthesia group (%)	Incisal anesthesia group (%)	Block anesthesia group (%)
Tongue	100	6.7	66.7
Upper Lips	78.6	46.7	60.0
Lower Lips	64.3	13.3	66.7
Cheeks	85.7	6.7	86.7
Upper Teeth	21.4	86.7	73.3
Lower Teeth	14.3	80.0	66.7
Palate	7.1	0	6.7
Others	14.3	6.7	6.7

## Hold force, split force, and duration of split

**Fig. 2:**
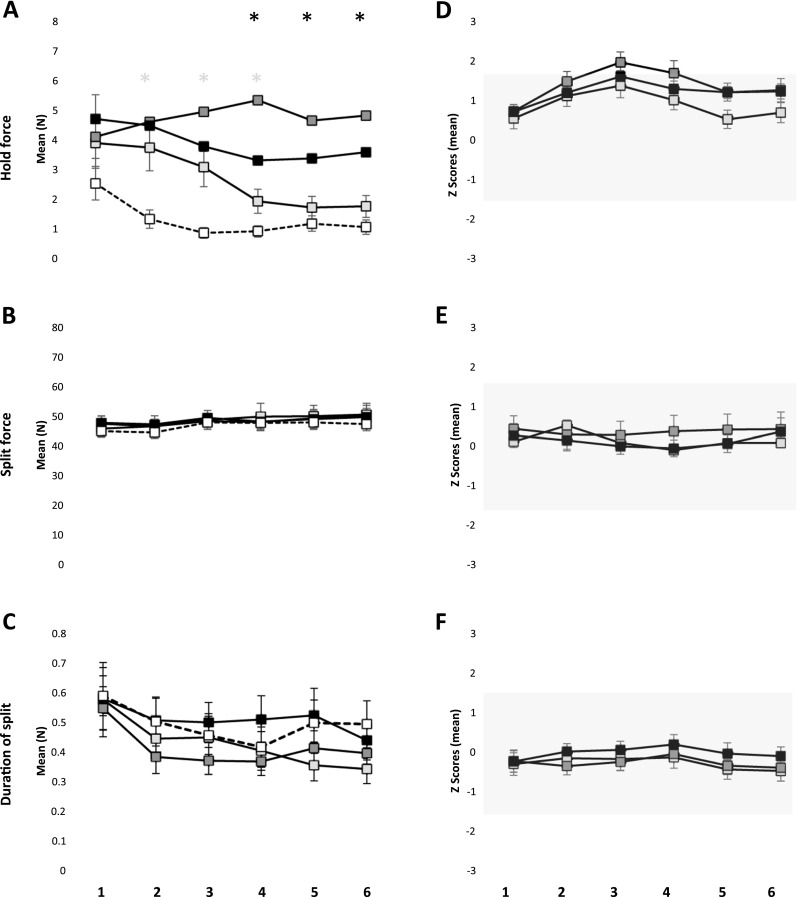
Mean ± standard error of mean hold force (**a**), split force (**b**), and duration of split (**c**) during six series of the behavioral task in the mucosal anesthesia, incisal anesthesia, block anesthesia, and the reference group. The *asterisk* denote significant difference in the mucosal anesthesia (*white*) and the block anesthesia (*black*) compared to the reference group (*P* < 0.05). Mean ± standard error of mean of the *Z* scores for hold force (**d**), split force (**e**), and duration of split (**f**) during six series of the behavioral task in the mucosal, incisal, and block anesthesia group. The *gray shades* denote 90 % confidence interval (−1.64 to 1.64)

The mean hold force was significantly higher in the incisal and block anesthesia group in comparison to the reference group (*P* < 0.001) (Fig. 2a). However, there was no significant difference in the mean hold force between the mucosal anesthesia and the reference group (*P* = 0.138). There was a significant effect of series (*P* < 0.001) and a significant interaction between the group and the series (*P* < 0.001) on the mean hold force. Post hoc analysis for series showed that hold force during the third to sixth series was significantly lower than the first series (*P* < 0.003) and hold force during the fourth to sixth series was significantly lower than the second series (*P* < 0.002). Post hoc analysis of interaction showed that the hold force during the second to fourth series in the mucosal anesthesia group was significantly higher than the second to fourth series in the reference group (*P* < 0.004) and fourth to sixth series in the mucosal anesthesia group was significantly lower than the fourth to sixth series in the incisal and block anesthesia group (*P* < 0.017). Furthermore, the hold force during all the six series in the incisal and block anesthesia group were significantly higher than the reference group (*P* = 0.004).

There was no significant effect of groups on either the mean split force (*P* = 0.975) or the mean duration of split (*P* = 0.486) (Fig. 2b, c). However, there was a significant effect of series on both the mean split force (*P* = 0.003) and the mean duration of split (*P* < 0.001). Post hoc analysis of series showed that the mean split force during the fifth and sixth series was significantly higher than the second series (*P* < 0.034). Post hoc analysis of series showed that the mean duration of split during the second to sixth series were significantly shorter than the first series (*P* < 0.001).

It was observed that the mean *Z* scores of hold force during the third and fourth series in the incisal anesthesia group were outside the range of −1.64 to 1.64 indicating a robust increase in the magnitude of hold force in the respective groups due to local anesthesia; see gray shade in (Fig. 2d). However, the mean *Z* scores of split force and the mean *Z* scores of the duration of split force were within the range of −1.64 to 1.64 indicating more subtle changes (Fig. 2e, f).

## Variability of hold force, split force, and duration of split force

**Fig. 3:**
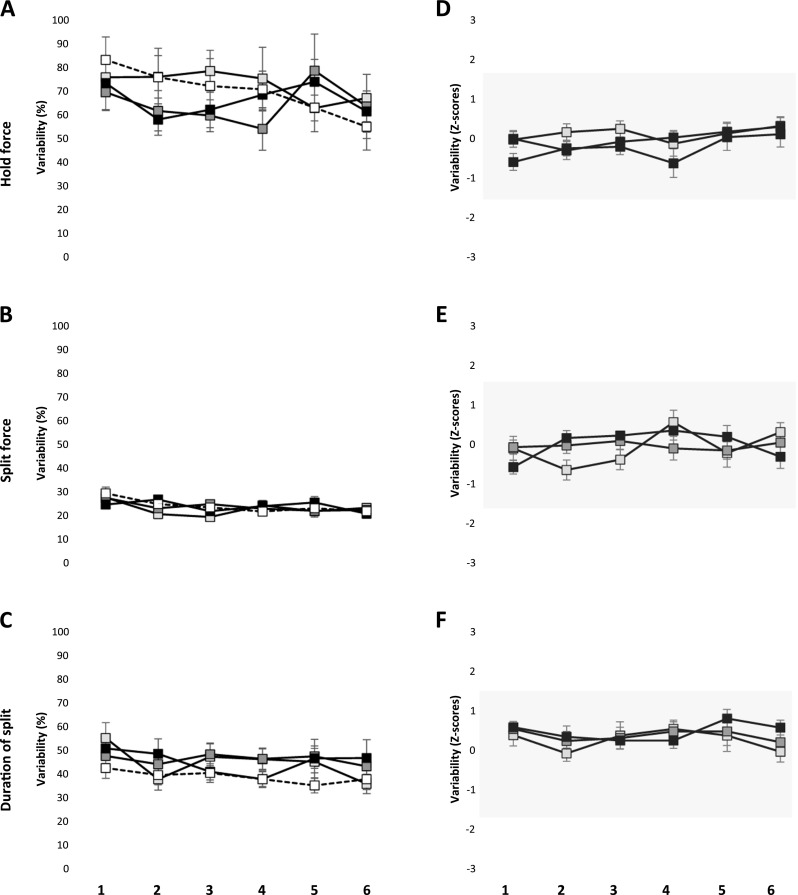
Mean ± standard error of mean of variability (expressed as co efficient of variation) in hold force (**a**), split force (**b**), and duration of split (**c**) during six series of the behavioral task in the mucosal anesthesia, incisal anesthesia, block anesthesia, and the reference group. Mean ± standard error of mean of variability of the *Z* scores for hold force (**d**), split force (**e**), and duration of split (**f**) during six series of the behavioral task in the mucosal, incisal, and block anesthesia group. The *gray shades* denote 90 % confidence interval (−1.64 to 1.64)

The variability of the hold force did not show any significant effect of groups (*P* = 0.677) but there was a significant effect of series (*P* = 0.014) (Fig. 3a). Post hoc analysis revealed that the variability of hold force in the sixth series was significantly lower than the first series (*P* < 0.003). Similarly, the variability of split force did not show any significant effect of groups (*P* = 0.641) but there was a significant effect of series (*P* < 0.001) (Fig. 3b). Post hoc analysis revealed that the variability of split force in the third to sixth series was significantly smaller than the first series (*P* < 0.014). There was an overall significant effect of groups (*P* = 0.039) and a significant effect of series (*P* = 0.035) on the variability of duration of split (Fig. 3c). However, post hoc test did not show any significant differences between the groups (*P* > 0.056), while the post hoc tests for series showed that the variability of duration of split during the sixth series was significantly lower than the first series (*P* = 0.022). Yet, the variability of the *Z* scores for the hold force, split force, and the duration of split force were within the range of −1.64 to 1.64 indicating subtle changes (Fig. 3d–f).

## EMG activity

### Hold phase

**Fig. 4:**
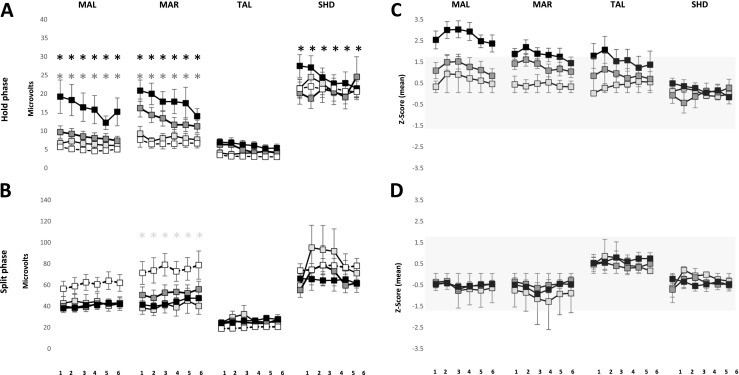
Mean ± standard error of mean of electromyographic activity (**a**, **b**) for right and left masseter (MAL and MAR); anterior temporalis (TAL) and suprahyoid (SHD) muscle in the mucosal anesthesia, incisal anesthesia, block anesthesia and the reference group during the hold phase (**a**) and split phase (**b**). The *asterisk* denote significant difference in the incisal anesthesia (*white*) and block anesthesia (*black*) compared to the reference group (*P* < 0.05). Mean ± standard error of mean of *Z* scores of electromyographic activity of right and left masseter (MAL and MAR); anterior temporalis (TAL) and suprahyoid (SHD) muscle in the mucosal anesthesia, incisal anesthesia, and block anesthesia during the hold phase (**c**) and split phase (**d**). The *gray shades* denote 90 % confidence interval (−1.64 to 1.64)

The mean EMG activity of MAL and MAR during the hold phase showed a significant effect of groups (*P* < 0.001) and series (*P* < 0.001) with no significant interaction between groups and series for both MAL (*P* = 0.107) and MAR (*P* = 0.094) (Fig. 4a). Post hoc analysis of groups showed that the EMG activity was significantly higher in the incisal and block anesthesia group than the reference group both for MAL (*P* < 0.025) and MAR (*P* < 0.005). Further, EMG activity of the block anesthesia group was significantly higher than the mucosal and incisal anesthesia group (*P* = 0.039) for MAL and mucosal anesthesia group for MAR (*P* = 0.020). Post hoc analysis of series showed EMG activity during the third to sixth series was significantly lower than the first series for both MAL (*P* < 0.011) and MAR (*P* < 0.010). Further, EMG activity during the fourth to sixth series was significantly lower than the second series, for MAL (*P* < 0.003) and the sixth series was significantly lower than the first for MAR (*P* = 0.043). The EMG activity of TAL and SHD did not show significant effect of groups (*P* = 0.201; *P* = 0.479; respectively). However, there was a significant effect of series (*P* < 0.001) and a significant interaction between groups and series for TAL (*P* = 0.042) and a significant effect of series for SHD (*P* = 0.009). Post hoc analysis for series revealed that EMG activity during the fourth to sixth series was significantly lower than the first series (*P* < 0.014), and the sixth series was significantly lower than the second series for TAL. Similarly, post hoc analysis for series revealed that EMG activity during the fifth series was significantly lower than the second series (*P* < 0.017) for SHD. Further, post hoc analysis for interaction showed EMG activity of TAL during all the series in the block anesthesia group was significantly higher than the reference (*P* < 0.001) and mucosal anesthesia group (*P* < 0.061).

### Split phase

The mean EMG activity showed no significant effect of groups for MAL (*P* = 0.107), TAL (*P* = 0.391), and SHD (*P* = 0.335) (Fig. 4b). However, there was a significant effect of groups on the EMG activity of MAR (*P* = 0.011) and a significant effect of series on the EMG activity of MAR (*P* = 0.022) and SHD (0.019). Post hoc analysis of groups showed a significantly lower EMG activity of MAR in the mucosal anesthesia group as compared to the reference group (*P* = 0.006). Post hoc analysis of series for MAL did not reveal any significant differences (*P* > 0.060) whereas, post hoc analysis of series for SHD showed a significantly higher EMG activity during the third series than the first series, during the split phase.

The mean *Z* scores of the EMG activity during all the six series of MAL and the first five series of MAR along with the first and second series of TAL in the block anesthesia group were above the range of −1.64 to 1.64 indicating a robust increase in the EMG activity during the hold phase due to the block anesthesia (Fig. 4c). However, the mean *Z* scores of the EMG activity during all the series in all the three groups during the split phase were within the range of −1.64 to 1.64 indicating subtle changes due to local anesthesia (Fig. 4d).

## Variability of EMG activity

### Hold phase

**Fig. 5:**
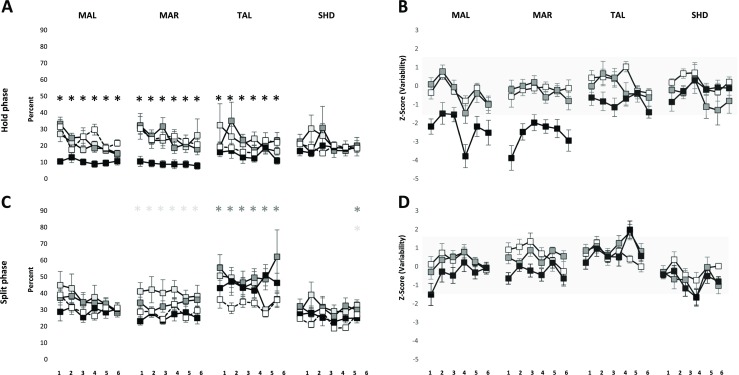
Mean ± standard error of mean of variability of electromyographic activity for right and left masseter (MAL and MAR); anterior temporalis (TAL), and suprahyoid (SHD) muscle in the mucosal anesthesia, incisal anesthesia, block anesthesia, and the reference group during the hold phase (**a**) and split phase (**b**). The *asterisk* denote significant difference in the mucosal anesthesia (*white*), incisal anesthesia (*gray*), and block anesthesia (*black*) compared to the reference group (*P* < 0.05). Mean ± standard error of mean of *Z* scores of electromyographic activity for right and left masseter (MAL and MAR); anterior temporalis (TAL) and suprahyoid (SHD) muscle in the mucosal anesthesia, incisal anesthesia, and block anesthesia during the hold phase (**c**) and split phase (**d**). The *gray shades* denote 90 % confidence interval (−1.64 to 1.64)

The variability of EMG activity showed significant effect of groups for MAL (*P* < 0.001), MAR (P < 0.001), and TAL (*P* = 0.016) but not for SHD (*P* = 0.206) (Fig. 5a). Post hoc analysis showed a significantly lower variability of EMG activity in block anesthesia group as compared to the mucosal, incisal, and the reference group (*P* < 0.001; *P* < 0.001, respectively) for MAL and MAR and a significantly lower variability of EMG activity in the block anesthesia group as compared to mucosal anesthesia and reference group (*P* < 0.041) for TAL. There was also a significant effect of series on the variability of EMG activity for MAL (*P* < 0.001) and MAR (*P* < 0.001), but not for TAL (*P* = 0.322) and SHD (*P* = 0.389). Post hoc analysis of series showed a significantly lower EMG activity during the third, fifth, and sixth series in comparison to the first series (*P* < 0.001) for MAL and the fourth to sixth series were significantly lower than the first series (*P* < 0.001) for MAR.

### Split phase

The variability of EMG activity showed significant effect of groups for MAR (*P* = 0.001) and TAL (*P* = 0.022) but not for MAL (*P* = 0.269) and SHD (*P* = 0.414) (Fig. 5b). Post hoc analysis showed that the variability of EMG activity was significantly higher in the mucosal anesthesia group as compared to the reference group (*P* = 0.021) and significantly lower in the block anesthesia group as compared to incisal and mucosal anesthesia group (*P* < 0.002) for MAR. Similarly, the variability of EMG activity of the incisal anesthesia group was significantly higher than the reference group (*P* = 0.018) for TAL. There was also a significant effect of series on the variability of EMG activity for MAL (*P* = 0.027) and SHD (*P* = 0.026). Post hoc analysis of series showed that the variability of EMG activity during the third series was significantly lower than the first series for MAL and the fourth series was significantly lower than the first series (*P* = 0.017), for SHD. The interaction between groups and series was also significant for SHD (*P* < 0.001). Post hoc analysis of interaction revealed that the fifth series in the mucosal and incisal anesthesia group was significantly higher than the fifth series in the reference group (*P* < 0.015).

The mean *Z* scores of the variability of EMG activity during the first and third to sixth series in the block anesthesia group for MAL and all the series in the block anesthesia group of MAR were above the range of −1.64 to 1.64 indicating robust increase in the EMG activity due to block anesthesia (Fig. 5c). The mean *Z* scores of the EMG activity during the fifth series in the incisal and block anesthesia group for TAL and the fourth series in the incisal and block anesthesia group for SHD were above the range of −1.64 to 1.64 indicating robust change in the EMG activity due to local anesthesia (Fig. 5d).

## Discussion

The present study assessed oral motor performance and behavioral adaptation after alteration of different orofacial afferent inputs, during a repeated hold-and-split oral fine motor task. The results of the study show an increase in the magnitude of the hold force due to the perturbation of the sensory inputs from the PMRs (incisal and block anesthesia group), as compared to a reference group (without anesthesia). There was also a significant increase in the EMG activity of the masseter muscles (MAR and MAL) due to incisal and block anesthesia and a significant increase in EMG activity of TAL due to block anesthesia, in comparison to the reference group; during the hold phase. Further, there was no significant effect of local anesthesia on the magnitude of split force but a significant decrease in the EMG activity of the MAR due to mucosal anesthesia, during the split phase. The results also reveal that there was no significant effect of local anesthesia on the variability of the hold and split force. However, there was a significant decrease in the variability of EMG activity of the jaw closing muscles (MAL, MAR, and TAL) in the block anesthesia group as compared to the reference group, during the hold phase. Further, there was a significant increase in the variability of EMG activity of MAR in the mucosal anesthesia group and a significant increase in the EMG activity of TAL in the incisal anesthesia group, compared to the reference group, during the split phase. The results also indicate the propensity of optimization in bite force values and jaw muscle activity due to repeated performance of the hold and split task. These findings, in general, may provide insights to the notion that neuroplasticity of face motor cortex occurs in association with changes in the oral environment, such as the loss or alterations of sensory inputs from dental or other oral tissues following prosthetic rehabilitation procedures.

There is substantial evidence from previous studies which confirms that cortical representations of body parts are continuously modulated in response to behavior learning and skill acquisition; for reviews, please see [16, 27]. In the event of injury or alteration in peripheral inputs either due to deafferentation (in animal models) or local anesthesia, there is reorganization of the central nervous system, which could be a useful model to study the short-term plasticity changes [16]. Further, adapting to an altered motor movement requires repetition of such movements. Hence, in the present study, it was decided to alter the peripheral orofacial sensory inputs (with local anesthesia) and further investigate the adaptation of the jaw muscles to the altered motor movement by repetition of the hold and split task.

## Effects of altering different orofacial afferent inputs

In the present study, we have compared the perturbation of the force and EMG activity under the influence of local anesthesia (mucosal, incisal, and block) to a reference group. The data for the reference group was obtained from our previous published study where 20 healthy participants performed 60 repetitions of the hold and split task in the exact same manner as the present study but without the influence of anesthesia [22]. Further, in order to obtain more precise results, we have normalized the data by standardizing the measurements into *Z* score (standard scores). *Z* score allows converting scores from different data sets into scores that can be accurately compared to each other. In the present study, we have also analyzed if the changes in the observed variables which are indications of perturbations due to local anesthesia are subtle or robust. Thus, a *Z* score of zero indicates that the score is the same as the mean whereas a positive or negative *Z* score is indicative whether it is above (gain/greater perturbation) or below (loss/lesser perturbation) the mean. We propose *Z* scores to evaluate perturbations in comparison to *healthy* individuals could be a novel way to study fine motor physiology. We also think that this method is a novel way of making meaningful comparisons across different data sets. Similar methods/analysis are used for the assessment of somatosensory function with quantitative sensory testing of healthy or orofacial, neuropathic pain patients; for example, please see [28, 29].

The results of the present study showed that the magnitude of the hold force was significantly higher in all the three anesthetized groups compared to the reference group. Further, the magnitude of hold force in the incisal and block anesthesia group was significantly higher than the mucosal anesthesia group (fourth to sixth series). It was also observed that the magnitude of EMG activity of the jaw closing muscles (MAL, MAR, and TAL) in the block anesthesia group was significantly higher than the reference group and the magnitude of EMG activity of the masseters (MAL and MAR) in the block anesthesia group was significantly higher than the mucosal anesthesia group during the hold phase. Therefore, it could be inferred from the results that mucosal anesthesia does not affect the EMG activity of the jaw closing muscles while anesthesia of the PMRs along with associated structures increases the EMG activity of the jaw muscles, during the hold phase. It was observed that there was a greater change in the magnitude of the hold force due to the disruption of the sensory inputs from the PMRs alone (incisal anesthesia group), as indicated by the positive *Z* scores above the 90 % (−1.64 and 1.64) CI. Further, the results also show more pronounced change in the EMG activity of the jaw closing muscles (i.e., MAL, MAR, and TAL) following total disruption of the sensory inputs from the jaw (i.e., block anesthesia group) as compared to disruption of PMRs alone (incisal anesthesia group) or oral mucosa alone (mucosal anesthesia group), during the hold as indicated by the positive *Z* scores above the 90 % CI. Hence, there is an increase (gain) in hold force and the corresponding EMG activity of the jaw closing muscles if there are alterations in sensory inputs from different orofacial mechanoreceptors. This also suggests that altering different orofacial afferent inputs would have different effects on oral fine motor control and jaw muscle activity.

The muscles spindles and temporomandibular joint (TMJ) receptors provide information about the relative positions of the jaws whereas, receptors in the periodontal ligaments, gingiva. and jaw bone provide information about the loading of the jaw and forces acting on the teeth. All of these receptor categories contribute to the fine coordination of the jaw muscles during biting or chewing [30]. It has been previously suggested that PMRs play a pivotal role in controlling and directing the force needed to hold the food morsel between the teeth and this control of forces is disrupted during periodontal anesthesia [5, 7]. In the present study, the mean hold force was highest in the incisal anesthesia group and lowest in the mucosal anesthesia group (among anesthetized groups) indicating greater perturbation in the magnitude of hold force or oral fine motor control due to reduced inputs from the PMRs (see Fig. 2a). Further, the EMG activities of the jaw closing muscles were also higher in the block anesthesia group (which included periodontal anesthesia) than the mucosal anesthesia group (see Fig. 4a). Moreover, the values of *Z* scores for hold force in the incisal anesthesia group (Fig. 2b) and EMG activity of MAL, MAR, and TAL in the block anesthesia group were >1.64 indicating a robust change in the magnitude of hold force due to reduced inputs from the PMRs as compared to oral mucosa alone. Hence, a reduced/loss of inputs from the PMRs (after incisal/block anesthesia) cannot be fully compensated by inputs from other orofacial mechanoreceptors (e.g., mechanoreceptors in muscle spindles or TMJ, etc.). The lack of peripheral afferent input (from PMRs) to the motor cortex downgrades the fine motor control of the jaws, for example, during a hold-and-split task as demonstrated in previous studies [5, 7, 23]. It was also previously suggested that the participants with a loss of PMRs, for example implant prosthesis patients, behave like participants with acute periodontal anesthesia in some aspects of biting behavior [5, 7, 31]. The participants with anesthesia similar to patients with implant supported prosthesis may rely (even though not full compensate) on distant orofacial mechanoreceptors (muscle spindle, TMJ, etc.) for sensory inputs which explains the relatively poor performance of fine motor tasks in these groups [31].

The magnitude of the split force, duration of split, and the jaw muscles activity (except for MAR) during the split phase did not show any significant effect of anesthesia. The results of the present study also show that the magnitude of split force and the duration of split did not differ greatly from the previous study [22] as indicated by the mean *Z* score, which was around zero (Fig. 2d). This finding that the split force did not seem to have been affected by the influence of anesthesia was similar to previous findings where no significant differences in the quantitative measurements of split force was observed when performing the hold and split task under the influence of anesthesia [5, 7, 11, 23]. Probably, the split force is mainly dependent on the factors reflecting the mechanical properties of food and the cleaving effects of the teeth or incisal edges and not by the presence or lack of sensory information from the PMRs [11, 23]. A previous study has reported no significant effect of anesthesia on the maximum voluntary bite forces and EMG activity [32]. However, it was observed that the magnitude of EMG activity of MAR was significantly lower in the mucosal anesthesia group than the reference group, during the split phase. Therefore, alteration of different orofacial afferent inputs may perturb both the “manipulative” (hold force) and “power” (split force) elements of some aspects of oral fine motor control [7].

## Perturbation of oral fine motor control

Variability has been established as a signature of skilled or rather unskilled motor performance [33]. Therefore, in the present study, we measured the variability as a measure of skilled performance. It has been suggested that anesthesia of the teeth blocks the signals from the PMRs and significantly elevates both the magnitude and the variability of the holding forces [5, 7, 8]. In the present study, we report an increase in the magnitude of the hold force (see discussed earlier) but no change in the variability of both the hold and split force under the influence of anesthesia. These comparisons may indicate that the relative precision of this oral fine motor task was not compromised in spite of anesthesia since the variability did not differ under the influence of anesthesia [5]. Further, even though there was a significant effect of groups on the variability of duration of the split yet post hoc analysis did not show any significant difference in groups compared to the reference group. This observation suggests that the participants were able to split the food morsel with the same duration irrespective of presence or absence of anesthesia. It was previously reported that anesthetizing the periodontium of the teeth resulted in a significant increase in the duration of split in peanuts (as food morsel) but not biscuits. This could indicate that the duration of split similar to the split force may also depend on the consistency/texture of the food morsel [11]. The variability of EMG activity was significantly lower (contrary to our hypothesis) in the block anesthesia group than the reference group for the MAL and MAR and TAL, during the hold phase. However, the variability of EMG activity was significantly higher in the mucosal anesthesia than the reference group for MAR and significantly higher in incisal anesthesia than reference group for TAL during the split phase. Moreover, the negative *Z* scores of the variability of EMG activity during the split phase show that there was a robust *decrease* in the EMG activity during the block anesthesia as compared to without anesthesia (reference group). Therefore, reduced/no afferent inputs from the orofacial/PMRs did not increase the variability of bite force values and jaw muscle activity; contrary to our hypothesis.

## Optimization of oral fine motor control

Skilled performance of a motor task is typically associated with a lower variability. Therefore, during repeated performance of a task variability undergoes changes leading to an overall reduction [33]. The results of the present study reveal that there was a significant effect of series on the variability of hold and split force; and the duration of split. The EMG activity of MAL and MAR during the hold phase and MAL during the split phase also showed significant effect of series. The post hoc analysis of the series have shown a decrease in all the significant parameters during the subsequent series in comparison to the first series, indicating signs of optimization of bite force values and jaw muscle activity; in spite of the reduced inputs from the orofacial/PMRs. However, since there were no significant interactions between series and groups, we cannot affirmatively say whether the optimization occurred in all three intervention groups or one of the groups including the reference group. Nevertheless, the results suggest the propensities for optimization in terms of decreased variability of bite force values and jaw muscle activity due to repeated performance of the hold and split task.

It has been suggested that reduced sensory feedback does not inhibit but rather hinders or perturbs motor learning [34]. Recent studies in rats have shown neuroplastic changes of motor representations in the face sensorimotor cortex within the first week after dental extraction [35]. Further, transcranial magnetic stimulation studies in humans have shown neuroplastic changes in the face motor cortex following interruption of sensory inputs induced by local anesthesia of orofacial tissues [34, 36]. As stated, the sensorimotor regulation during masticatory movements depends on the information from a variety of sense organs to regulate masticatory movements by the central nervous system [1]. By virtue of their location in the ligaments anchoring the tooth to the alveolar bone, the PMRs play a central role in encoding relevant aspects of forces acting on the dentition [37]. Therefore, they are likely to contribute tremendously to the generation and regulation of masticatory forces in various oral manipulative tasks.

Methodological considerations and limitations pertaining to the experimental design must be acknowledged. In order to obtain more precise results, it could be argued that while three types of anesthesia were employed, there was no control series where participants performed a series of the task without anesthesia. Although, having a control series may be important in determining that the participants were equal in physiological condition of jaw movements, we have previously observed and reported that majority of the changes have been observed during the first series [22]. Hence, we did not want to jeopardize or contaminate the participants with training effects (previous experience) and therefore, we did not allow practice trails before the start of the experiment. One of the measurements for determining the precision of task performance would be to evaluate the variability. Further, if two groups have to be compared for precision. we would need to compare the variability of the two groups. In the present study, we wanted to compare the perturbation caused by altering the peripheral sensory inputs (in the present study) to a control group [22] and therefore, we think that presenting the *Z* scores of the variation could be one new and valuable way of looking at the data.

The participants confirmed the subjective symptoms related to anesthesia; yet variability in the onset and duration of effect of local anesthesia, interindividual variability or inaccuracy of the injection technique, in some participants cannot be completely ruled out. The use of mepivacain 1 % without vasoconstrictor can be a methodological concern since the duration of action of mepivacain is short and the anesthetic effect is mild. It could be argued that the numerous differences between the series might be due to the increase and/or subsidence of the poor and short anesthetic effect. However, we wanted to retain the duration of action of the local anesthesia almost similar in all the three groups (especially mucosal anesthesia) and therefore, mepivacain would be a better choice. Nevertheless, there could be inconsistency in the spread of sensory blockade after local anesthesia and perhaps variability in the effect of the local anesthetic dose among participants. However, we think that since the data have also been presented in the form of *Z* scores which are a conservative method of data processing, these confounding factors would probably have no robust effect on our results. Further, even though surface EMG technique is a useful tool to study muscle physiology, it often has the inherent disadvantage of cross-talk between neighboring and underlying deep muscles especially when recording the EMG activity from the suprahyoid muscles. However, it was previously suggested that co-contraction of the suprahyoid muscles (e.g., digastric) may be greater in position-controlled tasks than compared to force-controlled tasks [38]. Since the present study was more related to force-controlled task, we believe that cross-talk effect would probably have influenced minimum effects on the outcomes.

## Conclusion

The results of the present study indicate that altering different orofacial afferent inputs may have different effects on some aspects of oral fine motor control. Further, reduced/no afferent inputs from the orofacial/PMRs did not increase the variability of bite force values and jaw muscle activity; indicating that the relative precision of the oral fine motor task was not compromised inspite of the anesthesia. The results also suggest the tendency of optimization of bite force values and jaw muscle activity due to repeated splitting of the food morsels inspite of alteration of sensory inputs. Previous studies have extensively demonstrated tissue reactions, occlusal features, bio-mechanics, and benefits of prosthodontics restorative, rehabilitative procedures [39]. However, recently, the fundamental issues like neural changes that occur when the stomatognathic system is altered (e.g., tooth loss, intraoral pain) and the neural processes underlying oral rehabilitative processes have received attention [39]. A complete and successful prosthodontic rehabilitation could probably only be achieved when the prosthesis is able to generate the sense of body ownership, i.e., when it is recognized as a part of his/her body scheme in prosthodontic patients. We believe that a sound understanding of these neural mechanisms may optimize clinical oral rehabilitation procedures and restore orofacial sensorimotor functions and thus enhance “ownership of the prosthesis” in prosthodontic patients [40, 41].
